# 171. Diagnostic stewardship to limit repeat plasma cytomegalovirus viral load testing

**DOI:** 10.1093/ofid/ofac492.249

**Published:** 2022-12-15

**Authors:** Akeatit Trirattanapikul, Ekawat Pasomsub, Sukanya Siriyotha, Oraluck Pattanaprateep, Angsana Phuphuakrat

**Affiliations:** Division of Infectious Diseases, Department of Medicine, Faculty of Medicine Ramathibodi Hospital, Mahidol University, Thailand, Krung Thep, Krung Thep, Thailand; Department of Pathology, Faculty of Medicine Ramathibodi Hospital, Mahidol University, Bangkok, Thailand, Krung Thep, Krung Thep, Thailand; Department of Clinical Epidemiology and Biostatistics, Faculty of Medicine Ramathibodi Hospital, Mahidol University, Bangkok, Thailand, Krung Thep, Krung Thep, Thailand; Department of Clinical Epidemiology and Biostatistics, Faculty of Medicine Ramathibodi Hospital, Mahidol University, Bangkok, Thailand, Krung Thep, Krung Thep, Thailand; Ramathibodi Hospital, Mahidol University, Bangkok, Krung Thep, Thailand

## Abstract

**Background:**

Serial monitoring of plasma cytomegalovirus (CMV) viral load monitoring with intervals of less than five days or using different assays for monitoring may cause unnecessary budgets for laboratory testing without changes in treatment, morbidity, and mortality.

**Methods:**

The pre-intervention retrospective study and post-intervention prospective cohort study were performed. In 2021, the inpatient electronic pop-up and telephone interview and feedback were used to limit unnecessary plasma CMV viral load testing. The rate of plasma CMV viral load testing being performed with intervals of less than five days was compared before and after protocol implementation using the Poisson regression model. The cost-effectiveness of plasma CMV viral load testing after protocol implementation was also studied.

**Results:**

After protocol implementation, there was a significant decrease in the rate of plasma CMV viral load test requests with intervals of less than five days from 11.8% to 6.2% [Incident rate ratio (IRR) 0.50, p-value < 0.001]. Of these, 38.9% was due to unintentional requests. After telephone interviews the rate of plasma CMV viral load test requests with intervals of less than five days decreased further to 4.7% (IRR 0.37, p-value < 0.001). The costs of plasma CMV viral load testing performed with intervals of less than five days and anti-CMV drugs were reduced significantly. (822,500 to 345,000 Thai Baht, p < 0.001 and 12,327,436 to 7,860,187 Thai Baht, p = 0.001)

Incidence of plasma CMV viral load testing performed with intervals of less than five days

**Figure:**
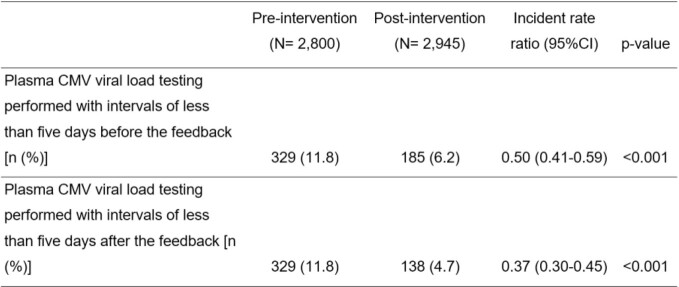

Costs of plasma CMV viral load testing, anti-CMV drug, bronchoscopy, and gastrointestinal endoscopy

**Figure:**
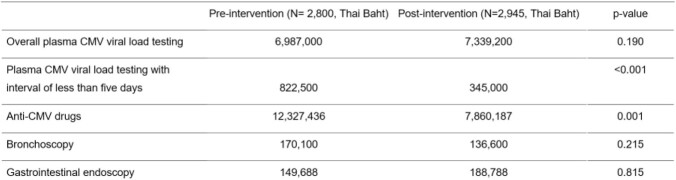

**Conclusion:**

The diagnostic stewardship program is helpful to reduce unnecessary plasma CMV viral load testing and costs without increasing CMV viremia and CMV diseases. This program should be maintained, and an electronic hard stop alert program should be developed.

**Disclosures:**

**All Authors**: No reported disclosures.

